# Evaluation of Diplopia Secondary to Seton Implantation Surgery: Ahmed Glaucoma Valve and Diplopia

**DOI:** 10.4274/tjo.galenos.2020.12269

**Published:** 2021-02-25

**Authors:** Sariye Taşkoparan, Osman Bulut Ocak, Semih Çakmak, Işıl Başgil Paşaoğlu, Birsen Gökyiğit, Banu Solmaz

**Affiliations:** 1 University of Health Sciences Turkey, Beyoğlu Eye Health Application and Research Center, Department of Ophthalmology, İstanbul, Turkey

**Keywords:** Diplopia, strabismus, Ahmed glaucoma valve

## Abstract

**Objectives:**

To determine and evaluate the rate of diplopia after Ahmed glaucoma valve (AGV) implantation surgery.

**Materials and Methods:**

The records of patients who underwent AGV implantation in our hospital between the years of 2010 and 2017 were retrospectively reviewed. Patients who were referred to our strabismus department due to binocular diplopia after AGV implantation surgery were included. The details of postoperative day 1, day 7, day 15, and 1 month examinations were recorded. In the postoperative period, the onset time of diplopia complaints, diplopia type, and the presence of diplopia at distance and near fixation were noted. Ocular motility examination and deviation measurements were evaluated.

**Results:**

Ten (47%) of 211 patients who underwent AGV implantation in our hospital between 2010 and 2017 met the study inclusion criteria. Six of the 10 patients were men (60%) and 4 were women (40%). The mean age of the patients was 44.5 (34-63) years. Complaints of diplopia developed at a mean of 14.5±12.3 (1-30) days after AGV implantation. The prism measurements of the patients were found to be 8.4±1.4 prism diopters (PD) exotropia and 7.1±8.8 PD hypotropia. While 8 patients had diplopia only at near distance, 2 patients had diplopia at both distance and near. Three patients were treated with prismatic glasses, and their complaints of diplopia recovered spontaneously in 5.11±4.10 months. The other 7 patients were followed up without treatment, and their diplopia complaints resolved spontaneously in 6.11±4.40 months.

**Conclusion:**

Although most of the diplopia that develops after AGV implant surgery resolves without treatment, prismatic glasses might be considered as a treatment option in patients whose diplopia affects their daily lives.

## Introduction

The main goal of glaucoma surgery is to achieve target intraocular pressure (IOP) and slow the progression of glaucoma-related vision and visual field loss. Seton implantation is one of the most commonly performed procedures, especially in cases where trabeculectomy surgery has failed.^1^

The Ahmed glaucoma valve (AGV) is one of the most frequently used devices in seton implant surgery. AGV implantation is preferred in patients that are non-responsive to medical treatment and have not had successful outcomes from other glaucoma surgeries.^2^ As with any surgery, some complications may be observed after AGV implantation. Complications such as choroidal effusion, hyphema, shallow anterior chamber, suprachoroidal hemorrhage, vitreous hemorrhage, positive Seidel test, cataract, corneal edema, hypotony, tube erosion, and endophthalmitis may occur in the early postoperative period.^3,4^

Strabismus and diplopia are also among the known complications following glaucoma drainage implant (GDI) surgery.^5^ In the literature, prevalence rates between 2.1% and 77% have been reported for strabismus ^6,7,8^ and between 1.4% and 23% for diplopia.^9,10^ Although patients often recover spontaneously, cases requiring intervention have also been reported.^11,12^

The aim of this study was to determine the incidence of diplopia in patients who underwent AGV implantation.

## Materials and Methods

The files of patients who underwent AGV implantation in our hospital between 2010 and 2017 were screened retrospectively. Patients who were referred to our strabismus unit due to binocular diplopia following AGV implantation were included in the study. Patients who had diplopia prior to AGV implantation, other extraocular muscle and/or neurological pathologies that may cause binocular diplopia, or monocular diplopia were excluded from the study.

From the records of the patients included in the study, we recorded their epidemiological characteristics and the distance and near best-corrected visual acuity (BCVA), IOP values, and biomicroscopic and fundus examination results of the eye to undergo AGV implantation, measured at the last preoperative examination. Distance BCVA was evaluated using a Snellen chart at a distance of 6 m, and near BCVA was evaluated using a near reading chart developed by Eğrilmez et al.^13^ at a distance of 30 cm. BCVA values were converted to logMAR for statistical analysis. IOP values measured by applanation tonometry and detailed optic disc head examinations were noted from the patient records.

### Surgical Technique

The Ahmed FP7 (New World Medical Inc., Rancho Cucamonga, CA) device was used as the AGV implant.

A fornix-based flap of the conjunctiva and Tenon’s capsule was raised in the superotemporal quadrant. Before implantation, the system was primed by injecting balanced salt solution through the tip of the tube using a 26-gauge (G) blunt cannula, and the valve was checked. The tube body was then sutured to the sclera 8-10 mm from the limbus. The tube was trimmed, bevel up, to a length not exceeding the pupil margin and inserted into the anterior chamber through an incision made 2-3 mm from the limbus using a 22G needle. The tube was secured to the sclera at two points using 10/0 nylon sutures. The tube was covered with pericardium, which was sutured to the sclera with 10/0 nylon sutures. The conjunctiva was closed with 10/0 nylon sutures.

Any complications that occurred perioperatively were recorded. The findings from detailed examinations performed on postoperative day 1, day 7, day 15, and 1 month were recorded. For patients with complaints of diplopia, the time from surgery to diplopia onset, type of diplopia, and presence of diplopia at distance and near. Ocular motility examination findings and deviation measurements were noted. Using the duction grading described by Scott and Kraft ^14^ gaze limitations were classified as 0 (normal), -1 (up to 75% of full rotation), -2 (up to 50% of full rotation), -3 (up to 25% of full rotation), or -4 (up to midline). For patients with visual acuity worse than 0.7 logMAR, the angle of deviation was determined by distance and near Krimsky prism test using prism bars due to inadequate fixation of the eye. For patients with visual acuity better than 0.7 logMAR, the angle of deviation was recorded using prism bars at distance and near fixation. Deviation angles were recorded in prism diopters (PD).

All patients’ results from the Hess test, distance Worth 4 dot test (6 m), and near Worth 4 dot test with appropriate refractive correction at 30 cm distance were recorded. Patients who could not perform the Hess test and the distant and near Worth 4 dot tests were noted.

Patients whose diplopia persisted or did not improve for more than 3 months were identified. The follow-up records of patients who underwent medical intervention were evaluated to determine whether the intervention was adequate and whether the diplopia persisted. We evaluated whether the patients’ complaints of diplopia changed during approximately 1 year of follow-up and the time of diplopia resolution was determined.

As per routine protocol, the patients were informed preoperatively about the possible risks of surgery, and verbal and written informed consent were obtained for the AGV implantation procedure. The study received ethics committee approval and was designed in accordance with the principles of the Declaration of Helsinki. Statistical analysis was not performed in this study.

## Results

Of the 211 patients who underwent AGV implantation in our hospital between 2010 and 2017, 10 patients (4.7%) presented to our strabismus unit due to binocular diplopia and met the study criteria. Of these, 6 patients were men (60%) and 4 were women (40%). The mean age was 44.5 (34-63) years. The preoperative mean distance BCVA was 0.81±0.27 logMAR, preoperative mean near BCVA was 0.9±0.35 logMAR, and mean IOP was 34.0±14.2 (26-46) mmHg. Postoperative mean distance BCVA was 0.90±0.41 on day 1, 0.87±0.45 on day 7, 0.82±0.52 on day 15, and 0.8±0.34 logMAR at 1 month. Postoperative mean IOP was 8.2±3.1 on day 1, 7.9±2.1 on day 7, 12.3±4.7 on day 15, and 12.4±5.6 mmHg at 1 month.

The Ahmed FP7 (New World Medical Inc., Rancho Cucamonga, CA) was used in all cases and was positioned in the superotemporal region between 10 and 11 o’clock in the right eye and between 1 and 2 o’clock in the left eye. The procedures were performed under general anesthesia and written and verbal consent was obtained from all patients preoperatively. The preoperative epidemiological and clinical findings of all patients are shown in Table 1.

**Figure 1:**
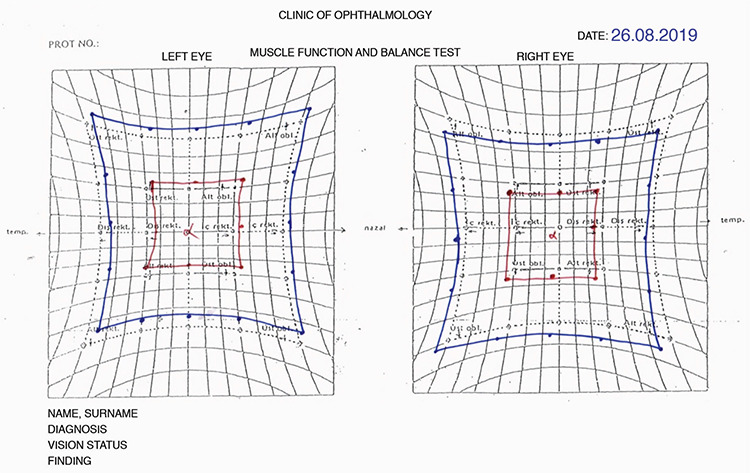
Patient 6, Hess test on postoperative day 14

**Figure 2:**
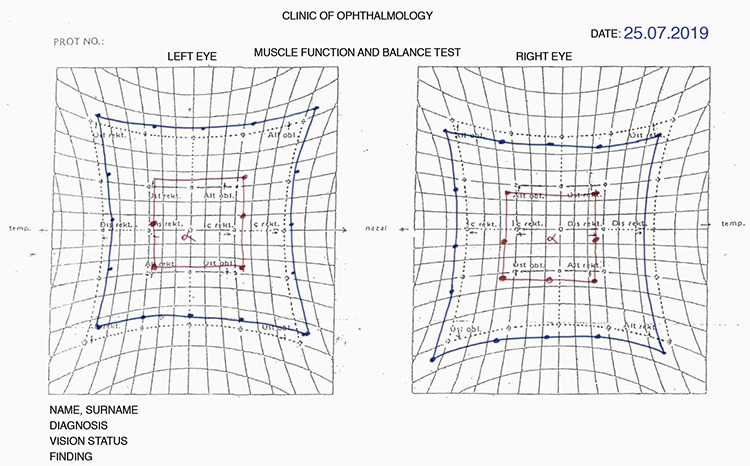
Patient 10, Hess test on postoperative day 18

**Figure 3:**
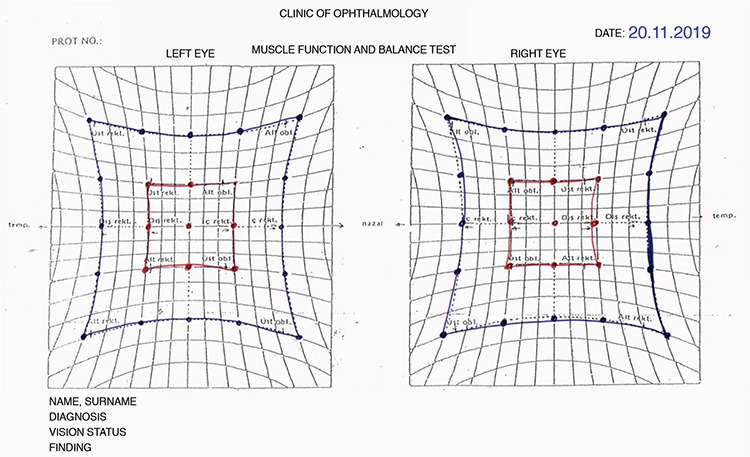
Patient 6, Hess test after diplopia resolved

**Figure 4:**
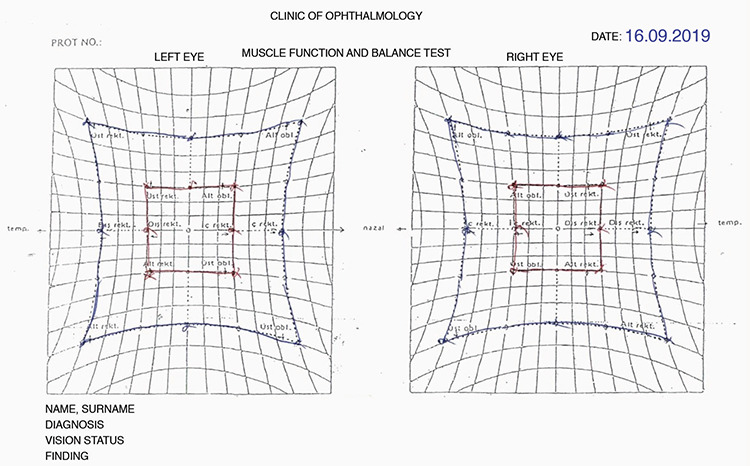
Patient 10, Hess test after diplopia resolved

Diplopia developed after a mean of 14.5±12.3 (1-30) days after AGV implantation. On ocular motility examination of these patients, -2 abduction limitation was observed in 4 patients, -2/-2 abduction/elevation limitation in 2 patients, -1/-2 abduction/elevation limitation in 1 patient, -2/-1 abduction/elevation limitation in 1 patient; and -2 elevation limitation was observed in 2 patients. The mean prism measurements of the patients were 8.4±1.4 PD exophoria and 7.1±8.8 PD hypotropia. Near diplopia alone was detected in 8 patients, while both distance and near diplopia were detected in 2 patients. The mean near visual acuity of the 10 patients with near diplopia was 0.8 logMAR. The visual acuity of the 2 patients with distance diplopia was 1.0 logMAR. Hess tests performed by these 2 patients during their complaints and after their complaints resolved are shown in Figures 1, 2, 3, and 4. All patients had vertical diplopia and none of the patients complained of horizontal diplopia. The ocular motility findings of all patients are shown in Table 2.

Three patients were treated with prism glasses and their diplopia complaints resolved spontaneously after a mean of 5.11±4.10 months. Seven patients were followed up without treatment and their diplopia complaints resolved spontaneously after a mean of 6.11±4.40 months.

## Discussion

GDIs are used in the treatment of patients with refractory glaucoma for whom trabeculectomy has failed or is believed to have a very low chance of success. The Molteno implant, AGV, and Baerveldt glaucoma implant (BGI) are among the implants used.^15^

As with any surgical procedure, some complications may occur after these implant surgeries. These complications can include cataract, corneal edema, hypotonia, tube erosion, hyphema, suprachoroidal hemorrhage, endophthalmitis, and diplopia.^1,3,4^

Diplopia and strabismus are complications that develop following GDI surgery.^4,9^ Strabismus and diplopia may result from a mass effect caused by a very large bleb, muscle tension, adipose tissue herniation, Faden effect due to scarring under the rectus muscles, and acquired superior oblique syndrome.^16^ There are various studies in the literature on diplopia after placement of a GDI (Molteno, Baerveldt).^11,17^

In their study evaluating diplopia after GDI surgery (Molteno, BGI, AGV), Abdelaziz et al.^11^ found that the incidence of diplopia within the first year was 1.4%. Their study also included diplopia due to other GDIs, and unlike our study, the implants were placed in different regions (inferotemporal, superonasal). Only 2 of the patients that developed diplopia had AGV implants. In this study, 17 of 32 patients who developed diplopia received prism glasses, while 13 patients did not undergo any treatment. In addition, 3 patients who did not respond to treatment with prism glasses were treated surgically.

In a retrospective study by Huang et al.^18^, diplopia was detected in 4 (2.5%) of 159 patients who underwent AGV implant surgery. Diplopia was corrected by surgical intervention in 3 of these 4 cases, while the AGV implant had to be removed in the other patient.

In addition, Ayyala et al.^10^ reported temporary diplopia in 4 (4.7%) of 85 patients who underwent AGV implant surgery, and 50% of these cases spontaneously resolved within 3 months after surgery.

Kartı et al.^19^ reported a case of acquired Brown’s syndrome following AGV implantation, and prism glasses were used to correct the patient’s diplopia.

In our study, correction with prism glasses was used in 3 of 10 patients who developed diplopia following AGV implantation, whereas 7 patients were followed up without any treatment. The patients treated with prism glasses were those who could not tolerate diplopia, and in a mean of 5 months, these patients no longer needed prism glasses and their diplopia resolved. We observed that the complaints of untreated patients could resolve spontaneously within a mean of 6 months.

There are also publications in the literature in which surgical methods were preferred for the treatment of diplopia after GDI surgery. Roizen et al.^12^ showed that restriction caused by the fibrous capsule around the implant was responsible for strabismus after GDI surgery. As surgical treatment, they performed adjustable suture surgery and capsule extraction.

In the present study, none of the patients underwent surgical procedures and we observed that their diplopia complaints resolved within a 1-year follow-up period. We believe that their diplopia may have been caused by intraoperative manipulation of the rectus muscles and restriction of rectus muscle action due to the implant itself. Therefore, we believe that if local edema resulting from manipulation resolves within the weeks or months after surgery, the diplopia may also resolve spontaneously.

### Study Limitations

The retrospective design of this study is one of its main limitations. Another limitation is the relatively small number of patients included in the study.

## Conclusion

In this study, the incidence of binocular diplopia following AGV implantation was 4.5%, and in all cases the diplopia resolved without the need for surgical treatment. The mean resolution time of diplopia was 6.5 months. Although most cases of diplopia after AGV implant surgery resolve without treatment, prism glasses may be considered as a treatment option in patients whose diplopia adversely affects their daily lives. In routine examination practice, diplopia and strabismus evaluation is not commonly performed for patients who have undergone and/or will undergo glaucoma surgery. However, a standard pre- and postoperative diplopia and strabismus examination may be beneficial for patients undergoing glaucoma surgery.

## Figures and Tables

**Table 1:**
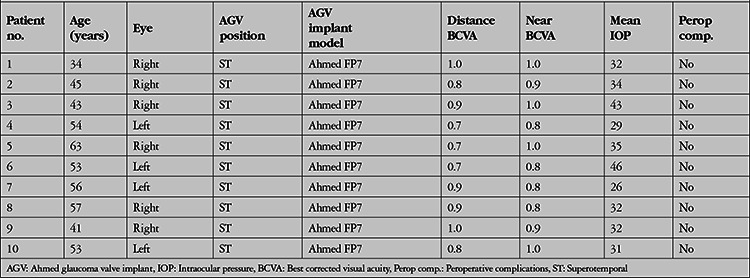
Epidemiological characteristics and preoperative clinical findings of the patients

**Table 2:**
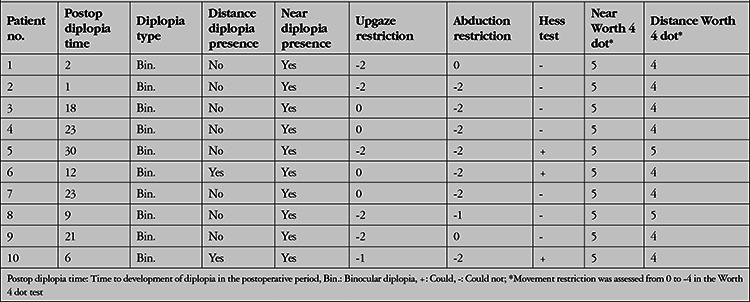
Postoperative ocular motility findings of the patients
